# Evaluation of surfactant proteins A, B, C, and D in articular cartilage, synovial membrane and synovial fluid of healthy as well as patients with osteoarthritis and rheumatoid arthritis

**DOI:** 10.1371/journal.pone.0203502

**Published:** 2018-09-20

**Authors:** Nadine Hartjen, Lars Bräuer, Beate Reiß, Horst Claassen, Stephanie Beileke, Fabian Garreis, Sebastian Hoogeboom, Michael Tsokos, Saskia Etzold, Brigitte Müller-Hilke, Kolja Gelse, Thomas Müller, Mary B. Goldring, Friedrich Paulsen, Martin Schicht

**Affiliations:** 1 Institute of Functional and Clinical Anatomy, Friedrich Alexander University Erlangen-Nürnberg (FAU), Erlangen, Germany; 2 Institute of Anatomy and Cell Biology, Martin Luther University Halle-Wittenberg (MLU), Halle (Saale), Germany; 3 Fraunhofer Institute for Supply Chain Services SCS, Nürnberg, Germany; 4 Institute of Legal Medicine and Forensic Sciences, Charité University Medicine Berlin, Berlin Germany; 5 Institute of Immunology, University of Rostock, Rostock, Germany; 6 Department of Trauma Surgery, Friedrich Alexander University Erlangen-Nürnberg (FAU), Erlangen, Germany; 7 Department of child and adolescent medicine, Pediatrics I, Pediatric Rheumatology, University of Halle-Wittenberg, Children's Hospital, Martin Luther University Halle-Wittenberg (MLU), Halle (Saale), Germany; 8 Department of Cell and Developmental Biology, Weill Cornell Medical College, New York, New York, United States of America; 9 Hospital for Special Surgery, HSS Research Institute, New York, New York, United States of America; Drexel University, UNITED STATES

## Abstract

**Objective:**

Surfactant Proteins (SPs) are well known from lung and form, along with phospholipids, a surface-active-layer at the liquid-air-interface of the alveolar lining. They play a major protective role by lowering surface tension, activating innate and adaptive immune defense at the lung mucosal interface, especially during infection. We analyzed the regulation of SPs in human and mouse articular chondrocytes, synoviocytes, and synovial fluid under healthy and inflammatory conditions, as well as in tissues of patients suffering from osteoarthritis and rheumatoid arthritis.

**Methods:**

Immunohistochemistry, RT-PCR, qRT-PCR, ELISA, Western blotting were performed in cell cultures and tissue samples to determine localization, regulation, and concentration of SPs.

**Results:**

All four SPs, were expressed by healthy human and mouse articular chondrocytes and synoviocytes and were also present in synovial fluid. Treatment with inflammatory mediators like IL-1β and TNF-α led to short-term upregulation of individual SPs *in vitro*. In tissues from patients with osteoarthritis and rheumatoid arthritis, protein levels of all four SPs increased significantly compared to the controls used.

**Conclusion:**

These results show the distribution and amount of SPs in tissues of articular joints. They are produced by chondrocytes and synoviocytes and occur in measurable amounts in synovial fluid. All four SPs seem to be differently regulated under pathologic conditions. Their physiological functions in lowering surface tension and immune defense need further elucidation and make them potential candidates for therapeutic intervention.

## Introduction

The major component of synovial fluid is the glycoprotein proteoglycan (PRG) 4 or lubricin. PRG4 is a surface-active mucinous glycoprotein secreted in the synovial joint that plays an important role for the frictionless gliding of diarthrodial joints [1]. The lubricating ability of the synovial fluid is thought to be due to surface-active phospholipids (SAPLs) that are part of the PRG4 fraction [1]. Synovial fluid contains two types of SAPLs, (1) phosphatidylcholines (PCs), which predominate and occur as saturated PCs and unsaturated species (USPCs), as well as (2) non-phosphatidylcholines (e.g., phosphatidyl glycerol), which are only present in small quantities. Saturated PCs are the dominant class of PC in the lung and, of these, dipalmitoyl phosphatidylcholine (DPPC) is the most surface-active component [1, 2]. DPPC together with the other lipids induces segregation of fluid-ordered and disordered phases in membranes and films at physiological temperatures. The segregation of the DPPC-enriched ordered phase has been associated with the ability of surfactant films to reduce surface tension, while the presence of two specific hydrophobic surfactant proteins (SP)-B and SP-C in the surfactant is absolutely required to facilitate surfactant dynamics, including film formation and re-spreading during lung expansion at inspiration [3, 4]. Thus, SAPLs can only fully function in the presence of SP-B and SP-C, as both act as an anchor between the phospholipid layer and the soluble phase, at least in the lung. Recent findings have shown that SP-B and SP-C make different contributions to inter- and intra-membrane lipid dynamics, and that their combined action could provide unique effects to modulate structure and dynamics of pulmonary surfactant membranes and films [5]. So far it is completely unknown whether SP-B and SP-C are part of diarthrodial joints.

Articular joint infection is a surprisingly rare event considering the frequency of joint arthrocentesis and other invasive procedures applied to limb joints and the restricted efficacy of the adaptive immune system in avascular tissues like cartilage and in T-lymphocytes undergoing hypoxia-related inhibition [6]. Recent studies have shown that articular joints are protected by a previously unrecognized defense mechanism in the form of endogenous antimicrobial peptide (AMP) production, e.g., defensins, RNAse 7, CAP37, cathelicidin LL37, and others that are constitutively produced by articular chondrocytes and synoviocytes and secreted into the synovial fluid, or—at least in some cases—can be induced by microbial invasion [7, 8]. Interestingly, upregulated gene expression of AMPs also occurs during osteoarthritis (OA) or rheumatoid arthritis (RA), resulting in increased synthesis of extracellular matrix (ECM)-degrading metalloproteinases and reduction in tissue inhibitors of metalloproteinases (TIMPs) 1 and 2. These findings implicate AMPs as multifunctional peptides with the ability to link host defense mechanisms and inflammation with tissue remodeling processes in articular cartilage [8]. AMPs are also active in pulmonary host defense, where they act together with non-surfactant collectins (for example avian collectins), as well as the two surfactant proteins SP-A and SP-D. These SPs belong to the family of c-type lectins capable of recognizing, inhibiting, and inactivating a broad spectrum of foreign pathogens, making them important effector molecules of the innate immune system [9, 10]. The presence of SP-A and SP-D has also already been demonstrated in synovial fluids of healthy horses [11], as well as in cultured cells isolated from the synovium (only SP-A) [12]. Both SP-A and SP-D have also been described in cases of RA [13, 14]. However, whether SP-A and SP-D contribute to immune response mechanisms in diarthrodial joints and whether they are also produced by articular chondrocytes has not yet been clarified.

Since SPs (A, B, C, and D) have been detected at several non-pulmonary sites in recent years and in different body fluids for example liquor [15], nasal mucosa [16] and testes [17], we hypothesized that SPs are produced not only by synovial membrane but also by articular chondrocytes and are secreted into the synovial fluid. On this basis, we performed a systematic analysis of all four SPs: SP-A, SP-B, SP-C and SP-D in human and murine knee joints. We then also tested whether cultured chondrocytes (primary cells and cell lines) are able to produce SPs *in vitro* and whether any induction of SP synthesis occurs under inflammatory stimuli or during joint diseases such as OA and RA. These investigations were performed in patient tissues, as well as in STR/ort mice, a mouse strain that is genetically predisposed to develop OA-like lesions in the medial tibial cartilage.

## Material and methods

### Tissue of human and mouse

Human cartilage and human synovium were obtained, with institutional review board approval of Charité Berlin, from patients aged 18–90 years in the Department of Legal Medicine, Charité Berlin, Germany (Tables 1 and 2). The study was approved by ethics Committee of the Medical Faculty of the Charité, Berlin. Tissues were dissected at least 10–14 h post mortem under cooling conditions. Half of each specimen was fixed in 4% paraformaldehyde for later paraffin embedding. The other half of each sample was frozen immediately at -80°C for use in molecular-biological investigations.

**Table 1:** Macroscopically healthy human articular cartilage.

age in years	18	22	28	33	38	41	48	54	59	62	69	73	83	90
**gender**	♀	♂	♂	♂	♂	♂	♂	♂	♂	♀	♂	♂	♀	♀

**Table 2:** Healthy human synovial membrane.

age in years	18	22	28	33	38	41	48	54	59	62	69	73	83	90
**gender**	♀	♂	♂	♂	♂	♂	♂	♂	♂	♀	♂	♂	♀	♀

Bronchial mucosa and lung samples served as positive control tissues. The tissue samples were obtained from cadavers (5 male, 11 female, aged 33–76 years) donated to the Department of Anatomy and Cell Biology, Martin-Luther-University Halle-Wittenberg, Germany. Experiments on human tissue were done in accordance with the provisions of the Helsinki Declaration for research involving human tissue. Prior to death, every donor bequeathed his/her body to the Institute for Anatomy and Cell Biology (MLU Halle-Wittenberg, Germany) for scientific and academic purposes by stating this in his/her last will.

Primary human chondrocytes were prepared from human knee cartilage (n = 3) immediately within one to two hours from patients that underwent total knee replacement and used to examine the expression and regulation of SPs after stimulation with inflammatory mediators in by real-time quantitative PCR (RT-qPCR). For culturing human chondrocytes, articular cartilage was obtained with institutional review board approval and informed consent prior to joint replacement surgery from three patients (aged 65–67 years) at the Department of Trauma Surgery of Friedrich Alexander University Erlangen-Nürnberg. Experiments were performed according to the Helsinki Declaration. Immediately after surgery in each case, cartilage specimens were transferred into sterile Hank’s Balanced Salt Solution (HBSS), Seromed L2035, to maintain pH and osmotic balance, as well as to provide the cells with water and essential inorganic ions and transported to the Nicolaus-Fiebiger-Centre for Molecular Medicine, Erlangen. In the laboratory, knee joint specimens were washed three times with a sterile HBSS and dissected under aseptic conditions. The harvested cartilage slices were washed three times in HBSS, Seromed L2035 containing 100 U/ml penicillin/streptomycin (Seromed A2210) and 2.5mg/ml amphotericin B (Seromed A2610). Slices of cartilage were incubated in 0.35% pronase (Boehringer 1459643)/HBSS for 60 min at 37°C, washed, and transferred overnight to a 0.05% collagenase solution (Sigma C-1889) in Hams F12 without phenol red (Seromed F0723). After centrifugation (400×g, 10 min) the cell pellet was resuspended in 10 ml medium containing serum (Hams F12), 10% fetal calf serum (FCS, Seromed S0115), 100 U/ml penicillin/ streptomycin, 2.5mg/ml amphotericin B, 1% ascorbic acid (10 mg/ml), 0,1% tocopherol (21.5 mg/ml)) and filtered through 20 μ nylon mesh. Vital cells in the final cell suspension were counted by the Trypan blue exclusion method with a hemocytometer.

Immortalized human cell lines (C28/I2 and T/C28a2) were obtained and used as described previously by Rösler et al. 2010 [18].

STR/ort mice are genetically predisposed to develop OA-like lesions of the medial tibial cartilage within six months of birth. Tissue slices from knee joints of STR/ort mice at different ages were used for hematoxylin and immunohistochemical staining, as described previously by Rösler et al. 2010 [18]. For age, sex, and grade of OA, see Table 3. In this study, knee joints from mice at the ages of 4, 9, 10 and 12 months were examined. These ages represent OA stages of 0° (4 month), I° (9 month), II° (10 month), and III° (12 month) (Table 3).

**Table 3:** Stage of osteoarthritis.

Grade of arthritis	age	gender
0	4 months	female
**I**	9 months	female
**II**	10 months	male
**III**	12 months	male
**IV**	>12 months	Female/male

Human synovial fluids (n = 12, healthy) were obtained, with institutional review board approval, from cadavers aged between 18–90 years at the Department of Legal Medicine, Charité Berlin, Germa (Table 4) within a postmortem interval of latest 14 hours under cooling conditions. Synovial fluid samples from OA and RA patients (n = 10 each), 8 male and 12 female, aged from 57 to 84, who underwent a total knee replacement were obtained within one to two hours from the Department of Molecular Immunology, Friedrich Alexander University Erlangen-Nürnberg (Table 5) during operation. Synovial membrane samples were obtained from RA patients undergoing clinically indicated synovectomy. Those from OA patients, as well as ten healthy control samples, were obtained during arthroscopies. OA synovial fluids were classified radiologically according to Kellgren and Lawrence [19] as late-stage OA. Those from RA patients were from early synovectomies, generally within 1–5 years from the onset of disease (Larson score 0–2).

**Table 4:** Healthy human synovial fluid.

age in years	40	46	49	49	50	58	58	60	64	67	67	68
**gender**	♂	♀	♂	♂	♂	♂	♀	♂	♂	♂	♂	♂

**Table 5:** OA and RA affected synovial fluid.

age in years	57	58	62	65	65	66	68	69	69	72	73	74	76	77	77	78	79	80	82	84
**gender**	♂	♀	♂	♀	♀	♀	♂	♂	♀	♀	♀	♀	♂	♀	♀	♀	♂	♂	♂	♀
**disease**	RA	RA	RA	RA	OA	RA	RA	OA	OA	OA	OA	RA	RA	RA	OA	RA	OA	OA	OA	OA

### Cell culture

Primary human articular chondrocytes from the tibial plateau of knees (n = 3) were seeded at a density of 50,000 cells/cm^2^ in 75cm^2^ flasks and cultured in DMEM/F-12 medium (Gibco 11039) containing 10% fetal calf serum (Seromed S0115), 100 U/ml penicillin/streptomycin (Seromed A2210), 1% ascorbic acid (10 mg/ml), 0,1% tocopherol (21.5 mg/ml in 1 ml ethanol) for about 5 days at 37°C and 5% O_2_. Medium was changed every third day. The cells were then washed with PBS and cultured in DMEM/F-12 medium without serum, containing, 100 U/ml penicillin/streptomycin, 1% ascorbic acid (10 mg/ml), 0,1% tocopherol (21.5 mg/ml in 1 ml ethanol), 1% pyruvate (Gibco 11360) and 1% cysteine (15.76 mg/ml) (Sigma C-1276) for 2-3h.

For culture in monolayer, chondrocytes were seeded at a density of 400,000-cells/ml medium/well in 75 cm^2^ flasks, and incubated for 5–7 days with medium containing serum (see above) at 37°C and 5% O_2_ with medium changes every third day, as described previously [20].

Incubations of cells with tumor necrosis factor α (TNF α), interleukin 1β (IL 1β), or a combination of both were performed to examine the effects of inflammatory mediators that are well known to play a major role in OA and RA. Cultured primary human articular chondrocytes and immortalized chondrocytic cell lines were changed to serum-free medium and then incubated with 10 ng/ml TNF α (Sigma Aldrich, St. Louis, USA) or 10 ng/ml IL 1 β (Sigma Aldrich, St. Louis, USA), alone or in combination, according to the scheme in Table 6. For this purpose, stock solutions of TNF α, IL 1β and TNF α + IL 1 β in serum-free medium were prepared and dissolved stepwise. Control cultures were incubated with serum-free medium only [20].

**Table 6:** Incubation with tumor necrosis factor α (TNF α), Interleucin 1 β (IL 1β) and a combination of both.

TNF α	0h	6h	12h	24h	72h
IL 1β	0h	6h	12h	24h	72h
TNF α + IL 1β	0h	6h	12h	24h	72h

### RNA isolation and complementary DNA (cDNA) synthesis

Total RNA was isolated from healthy synovial membrane samples (n = 15) and healthy stimulated primary chondrocytes from healthy donors (n = 3). Samples were frozen in liquid nitrogen and ground for 1–2 min with a Speedmill Plus apparatus (Analytik Jena AG, Jena, Germany). After precipitation with isopropanol and purification by repeated ethanol and DEPC H_2_O washes, RNA was isolated using the following protocol: contaminating DNA was eliminated by digestion of RNAse-free DNAse I (30 min at 37°C), which was then heat-inactivated after addition of EDTA 50 mM and incubation for 10 min at 65°C. For cDNA synthesis, isolated RNA was added with 1 μl oligo (dt)_15_ primer and 2 μg RNA. After centrifugation and incubation (5 min at 69°C), as well as addition of 4 μl 5x buffer, 2 μl 10 mM dNTP 0.5 μl RNase inhibitor 1 μl RevertAid H Minus Reverse Transcriptase and incubation for 60 min at 42°C and 10 min at 70°C, reverse transcriptase was inactivated and cDNA was finally generated. For each sample, materials from Invitrogen (Superscript™ II-RT-Kit) were used. To check the integrity of the translated/synthesized cDNA, glyceraldehyde-3-phosphate dehydrogenase (GAP-DH) was used as an internal control.

### Polymerase chain reaction (PCR)

The reaction mixture consisted of 2 μl cDNA, 2 μl 10 x PCR buffer, 2 μl 50 mM MgCl_2_, 2 μl dNTPs, 9.8 μl dH_2_O and 0.2 μl Taq DNA polymerase. The primer sequences used are listed in Tables 7 and 8. GAPDH was used as housekeeping gene. 35 cycles were performed for each primer pair. PCR conditions were as follows: (heat) pre-denaturation for 5 minutes at 95°C, 35 cycles of (heat) denaturation for 30 seconds at 95°C, annealing for 30 seconds at 56.5°C for SP-A, 60°C for SP-B and SP-D and GAPDH, and 55.8°C for SP-C, followed by an elongation step for 30 seconds at 72°C. Final extension was performed for 5 minutes at 72°C. As a negative control, cDNA was replaced by water. PCR products were detected by Thermo-Gradient-Cycler (Biometra GmbH Göttingen, Germany). All primers were synthesized by Metabion International AG, Martinsried, Germany.

**Table 7:** Primers used for RT-PCR (1μl each containing/presenting 10pmol). Metabion international AG, Martinsried, Germany.

Protein	Manufacturer	Annealing Temperature	Sequences 5´-3´	Bands
**SP-A** **(A2A4)**	Metabion	56.5°C	F:GATGGGCAGTGGAATGACAGG R:GGGAATGAAGTGGCTAAGGGTG	212bp
**SP-B** **(Mature Strict)**	Metabion	60°C	F: CACCATGTTCCCCATTCCTCT R:TCATCCATGGAGCACCGGAGGACGA	239bp
**SP-C** **Real 1**	Metabion	55.8°C	F:TCATCGTCGTGGTGATGGTG R:ATGGAGAAGGTGGCAGTGGTAA	110bp
**SP-D** **Real 1**	Metabion	60°C	F:TGCTGCTCTTCCTCCTCTCTGC R:GGGCGTTGTTCTGTGGGAGTAG	95bp
**GAP-DH**	Metabion	60°C	F: CCCTTCATTGACCTCAACTAC R: CCACCTTCTTGATGTCATCAT	300bp

**Table 8:** Primers used for qRT-PCR (1μl each containing/presenting 10pmol). Metabion international AG, Martinsried, Germany.

Protein	Manufacturer	Annealing Temperature	Sequences 5´-3´	Bands
**SP-A** **Real 2**	Metabion	60°C	F:CATTGCTGTCCCAAGGAATC R:CCGTCTGAGTAGCGGAAGTC	125bp
**SP-B** **Real 3 Roche**	Metabion	60°C	F:GGGTGTGTGGGACCATGT R:CGTCTCACTTGGCTTTTCCTT	101bp
**SP-C** **Real 2**	Metabion	60°C	F:ACATGAGCCAGAAACACACG R:GGAGAAGGTGGCAGTGGTAA	109bp
**SP-D** **Real 1**	Metabion	53°C	F:TGCTGCTCTTCCTCCTCTCTGC R:GGGCGTTGTTCTGTGGGAGTAG	125bp
**18S r RNA**	Metabion	60°C	F: GGTGCATGGCCGTTCTTA R: TGCCAGAGTCTCGTTCGTTA	110bp

### Real-time RT-PCR (qRT-PCR)

Gene expression was analyzed by quantitative real-time RT-PCR (qPCR) using a LightCyler480^®^ system (Roche). The PCR reaction contained 10 μL LightCycler480^®^ 5x probe mastermix, 0.25 μL each of primer and 2 μL each of cDNA and 7.1 μl nuclease-free water. In each 96-well plate qPCR was performed with one cycle for 5 min at 95°C, 55 cycles for 15 seconds at 95°C, 30 seconds at 60°C and 1 second at 72°C, to confirm amplification of specific transcripts. SPs and 18S primers as well as the corresponding UPL probes (Table 8) were generated by using the ProbeFinder^TM^ software (Version 2.04, Roche). A standard curve was generated by serial dilutions of cDNA from non-stimulated cells. To standardize mRNA concentration, the transcript level of the housekeeping gene small ribosomal subunit (18S rRNA) was determined in parallel for each sample, and relative transcript levels were corrected by normalization based on the 18SrRNA transcript levels. All qPCRs were performed in triplicate and the changes in gene expression were calculated by the ΔΔC_t_ method.

### Protein isolation/extraction

Samples were frozen in liquid nitrogen and after adding Triton-buffer (300 μl) and protease and phosphatase inhibitors (10 μl/1 ml each), the samples were homogenized (Speedmill Plus, Analytik Jena AG, Jena, Germany), followed by incubation for 30 min on ice, centrifugation and another incubation for 20–30 min at 4°C. The protein concentration was measured by the Bradford assay (Thermo Scientific Nanodrop 2000c, Wilmington, USA).

### Western blot analysis

For Western blot analysis, proteins were isolated from human cartilage samples (n = 15) and human synovial fluids (n = 15). Total proteins (SP-A, SP-B and SP-D 50 μg, SP-C 100 μg) were analyzed by Western blotting. Proteins were resolved by reducing 15% SDS-polyacrylamide gel electrophoresis, electrophoretically transferred at room temperature for 2h at 0.8 mA/cm2 onto 0.1 μm pore size nitrocellulose membranes, and blocked for 30 minutes in blotting solution (5% milk powder). Bands were detected with primary antibodies to SP-A (1:500), SP B (1:250), SP-C (1:400) and SP-D (1:500), secondary antibodies (goat anti-rabbit (SP-C) / goat anti-mouse (SP-A, SP-B, SP-D)) conjugated to horseradish peroxidase 1:5000 (SP-A, SP-B, SP-D), 1:4000 (SP-C) applying chemiluminescence (ECL-Plus; Amersham-Pharmacia, Uppsala, Sweden) (Table 9). Human lung was used as a control. The molecular weights of the detected protein bands were estimated by use of standard protein marker (Prestained Protein Ladder, Fermentas, St.-Leon Roth, Germany) ranging from 11 to 170 kDa.

**Table 9:** All antibodies mentioned were used for Western blot analysis as well as for immunhistochemistry as prescribed/specified by the manufacturer.

Protein	Manufacturer	Dilution	Technique
**SP-A**	1.AB: Chemicon Millipore, USA: AB 3270	1:500	Western Blot Analysis
	2.AB: DAKO Denmark: goat anti-mouse	1:5000	
**SP-B**	1.AB: Chemicon Millipore, USA: AB 3276	1:250	Western Blot Analysis
	2.AB: DAKO Denmark: goat anti-mouse	1:5000	
**SP-C**	1.AB: Chemicon Millipore, USA: AB 3786	1:400	Western Blot Analysis
	2.AB: DAKO Denmark: goat anti-rabbit	1:4000	
**SP-D**	1.AB: Chemicon Millipore, USA: AB 4083	1:500	Western Blot Analysis
	2.AB: DAKO Denmark: goat anti-mouse	1:5000	
**SP-A**	1.AB: Chemicon Millipore, USA: AB 3270	1:50	Immunhistochemistry
	2.AB: DAKO Denmark: rabbit anti-mouse	1:200	
**SP-B**	1.AB: Chemicon Millipore, USA: AB 3276	1:50	Immunhistochemistry
	2.AB: DAKO Denmark: rabbit anti-mouse	1:200	
**SP-C**	1.AB: Chemicon Millipore, USA: AB 3786	1:50	Immunhistochemistry
	2.AB: DAKO Denmark: goat anti-rabbit	1:200	
**SP-D**	1.AB: Chemicon Millipore, USA: AB 3434	1:50	Immunhistochemistry
	2.AB: DAKO Denmark: goat anti-rabbit	1:200	

### Enzyme-Linked Immunosorbent Assay (ELISA)

Human cartilage samples (n = 15) and human synovial fluid samples were analyzed by quantitative sandwich ELISA. Analysis was performed using ELISA-kits from USCN Life Science Inc. Wuhan, China (E90890Hu 96 Tests for SP-A, E91622Hu 96 Tests for SP-B, E91623Hu 96 Tests for SP-C and E91039Hu 96 Tests for SP-D). The absorbance was quantified at a wavelength of 405 nm and 450 nm and compared with standard series, and the protein concentrations were determined for each sample and calculated as ng/mg total protein.

### Immunohistochemistry

For immunohistochemistry, tissue specimens from articular joint of human (n = 3), mouse (n = 4) and human synovial membrane (n = 3) were embedded in paraffin, sectioned (5 μm) and dewaxed. Immunohistochemical staining was carried out with antibodies against SP-A (1:50, MAB 3270), SP-B (1:50, MAB 3276), SP-C (1:50, MAB 3876), and SP-D (1:50,MAB 3434). Antigen retrieval was performed by pretreatment with microwave heating for 10 min in citrate buffer (pH 6) and non-specific binding was inhibited by incubation with porcine normal serum (Dako) 1:5 in Tris-buffered saline (TBS). Each primary antibody (1:50) was applied overnight at 4°C. The secondary antibodies (1:200) were incubated at room temperature for at least 2 h. Visualization was achieved with aminoethylcarbazole (AEC) for at least 5 min. Red staining in areas within the tissue sections indicated a positive antibody reaction. After counterstaining with hematoxylin, sections were mounted with Aquatex (Boehringer, Mannheim, Germany). Two negative control sections were used in each case: one was incubated with the secondary antibody only, the other with the primary antibody only. Visualization was carried out with a Keyence Biorevo BZ9000 microscope.

### Statistical analysis

After evaluating values for normal Gaussian distribution by the Kolmogorov–Smirnov test, we performed one-way ANOVA statistical analysis. For the interpretation of the results we used the Games-Howell post-hoc test because Levene's test for homogeneity of variances revealed an inequality of variances. The relative gene expression was normalized to the reference gen and mean ± SEM is presented columns. For a better overview the controls were set to 1 and the gene expression ratio was set in relation to the control.

All bar charts were generated and analyzed with GraphPad Prism (version 5). Results are plotted as means ± SEM. P-values of less than 0.05 were considered to be statistically significant and marked by asterisks (*).

## Results

### Expression of surfactant proteins at the mRNA level

**Fig 1:**
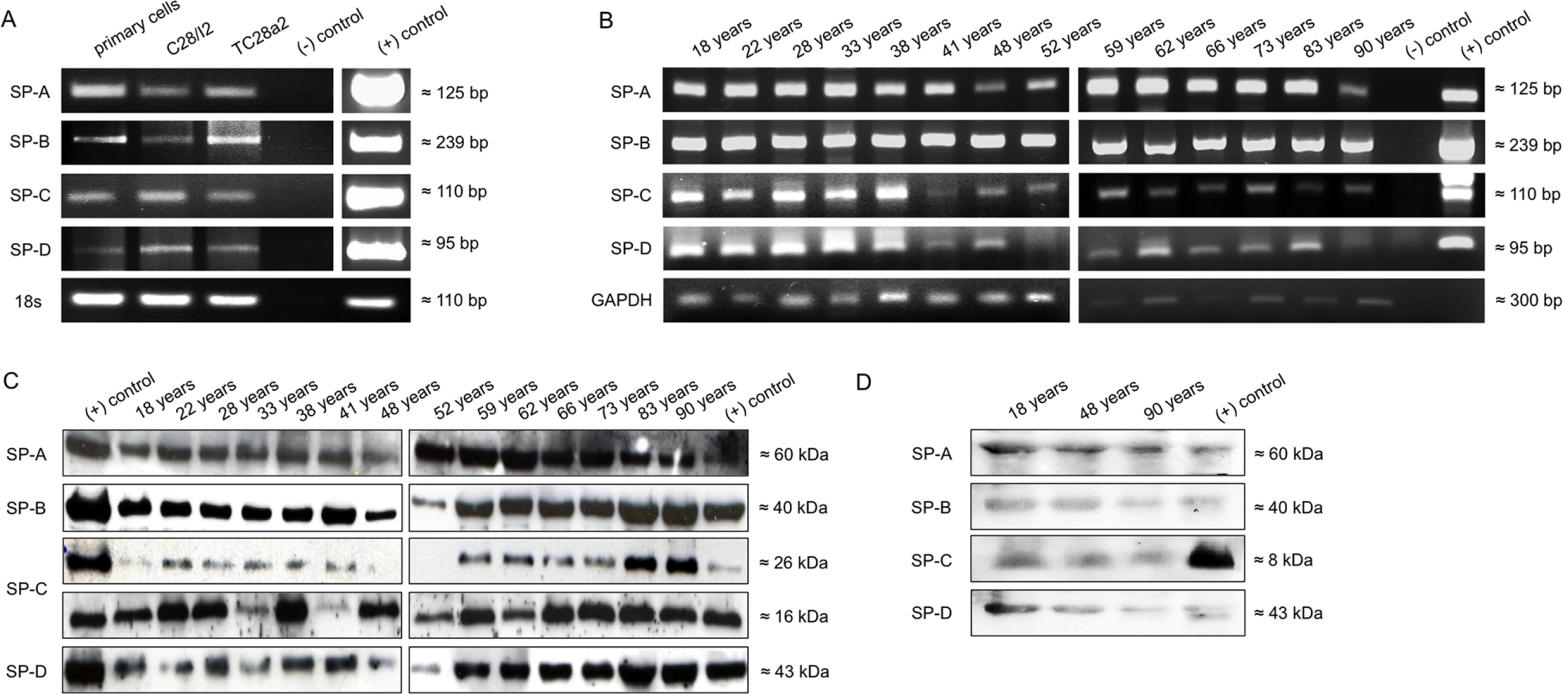
**Visualization of SP-A, SP-B, SP-C and SP-D by RT-PCR (A+B) and Western Blot (C+D). A**: SP-A, SP-B, SP-C and SP-D are detected by RT-PCR in primary chondrocytes and immortalized cell lines [C28/I2 and TC28a2] and **B**: synovial membrane from autopsied persons aged from18 up to 90 years, (-) negative control (water); (+) positive control, plasmid DNA carrying the full-length gene for the respective surfactant proteins. All RT-PCR analyses show cDNA amplification for the relevant surfactant protein in comparison to 18s or GAPDH. Western immunoblots of all surfactant proteins derived from the **C:** articular joint from autopsied persons aged from18 up to 90 years and **D:** synovial fluid from autopsied persons aged from 18; 48 and 90 years. The proteins were separated by SDS-PAGE under reducing conditions and show distinct bands for all four investigated surfactant proteins at the specific molecular weights (SP A: 60kDa, SP B: 40kDa, and SP D: 43kDa). For SP-C three visibly bands at 16kDa and 26kDa were detected for articular joint and 8kDa for synovial fluid. Lung was used as (+) positive control.

RT-PCR analyses of total RNA extracted from human primary chondrocytes, the immortalized chondrocytes cell lines, C28/I2 and T/C28a2, and tissue samples of human synovial membrane, revealed gene expression of all four SPs (SP-A: 120bp, SP-B: 239bp, SP-C: 142bp and SP-D: 95bp) (Fig 1A and 1B). For verification, plasmids containing the open reading frame for the corresponding surfactant protein were used as positive control. β-actin and GAPDH were used as loading control for RT-PCR.

### Synthesis of surfactant proteins at protein level

Western blots for SP-A, -B, -C, -D revealed that human cartilage (n = 15) and human synovial membrane (n = 3; between 18 and 90 years) synthesized SPs of the expected molecular weights (SP-A at ~60 kDa, SP-B at ~40 kDa, SP-C at ~26 kDa, ~16kDa, and ~8kDa, and SP-D at ~43 kDa (cf. Fig 1C and 1D). Lung tissue was used as positive control.

### Quantification of SPs in human cartilage and synovial membrane by ELISA

**Fig 2:**
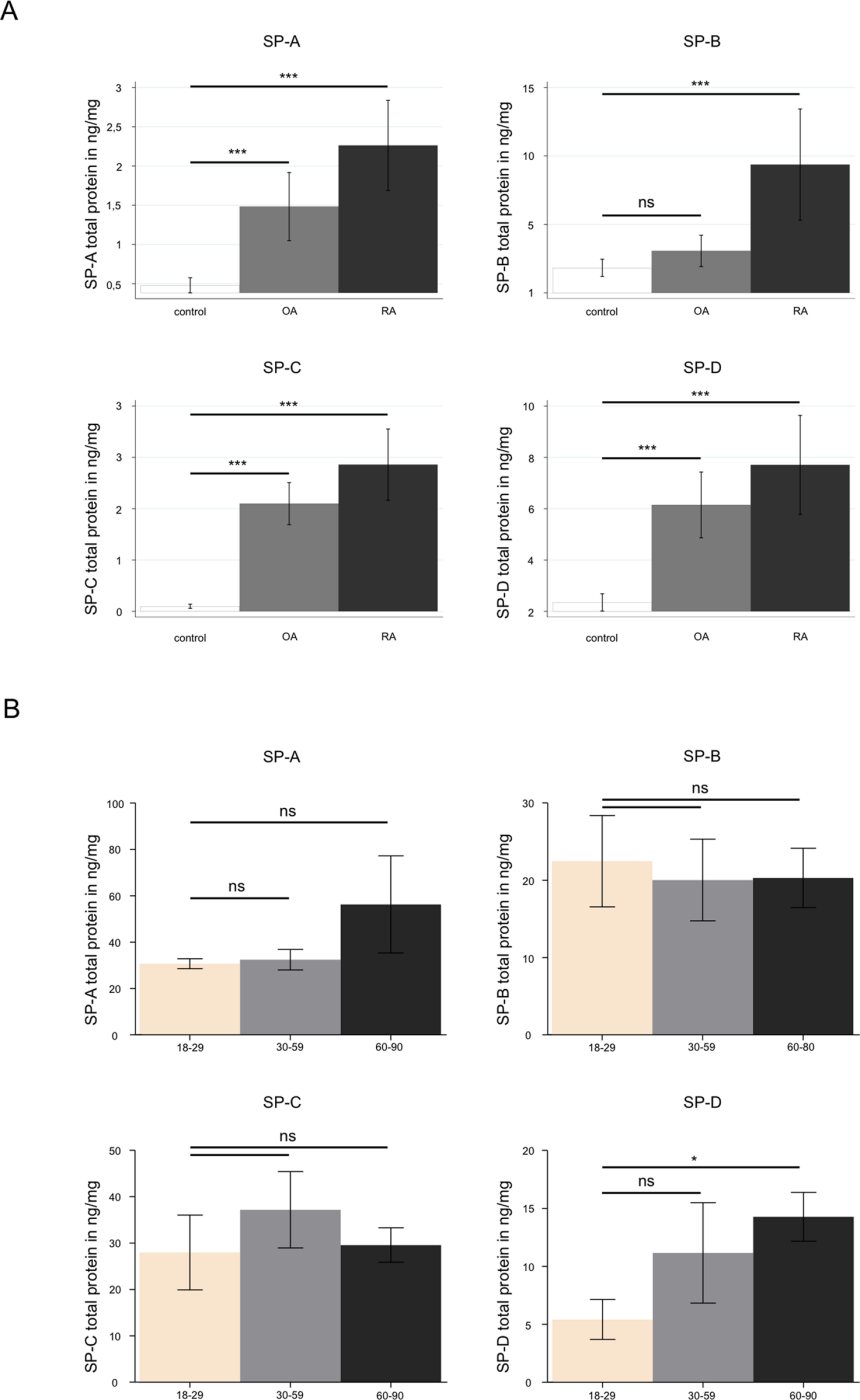
**Protein quantification of SP-A, SP-B, SP-C and SP-D: (A) in healthy human synovial fluid (HS) compared to synovial fluid of patients suffering from late stage osteoarthritis (OA) as well as rheumatoid arthritis (RA) and (B) in human articular cartilage from “healthy” cadavers. A:** ELISA of SP-A, SP-B, SP-C and SP-D derived from the following samples: healthy synovial fluid (control, n = 10); osteoarthritis (OA, n = 10) -and rheumatoid arthritis-affected synovial fluid (RA, n = 10). Mean values for all four SPs are significantly increased in OA and RA (significance p is shown in the figure for each surfactant protein). The protein concentration is expressed in ng/mg. Statistical significance: *P ≤ 0.05, **P ≤ 0.005, ***P ≤ 0.0005. **B:** ELISA analysis of SP-A, SP-B, SP-C and SP-D in human articular joint (n = 14), classified in groups depending on age: 0–30 years, 30–60 years and 60–90 years. The protein concentration is expressed in ng/mg. Statistical significance: *P ≤ 0.05, **P ≤ 0.005, ***P ≤ 0.0005.

ELISA quantification revealed significantly increased concentrations of SP-A, SP-B, SP-C, and SP-D in synovial fluids from patients suffering from OA (n = 10) and RA (n = 10), compared with synovial fluids from healthy people (HS) (n = 19) (cf. Fig 2A). These differences were statistically significant (***P ≤ 0.0005). All measured protein concentrations of the four SPs in samples with OA, RA or in healthy synovial fluid are given in S1 Table.

Quantification of SPs in human articular cartilage (n = 14) from patients aged between 18 and 90 years revealed no significant age-related change for SP-B and SP-C (cf. Fig 2B). In contrast, for SP-A and SP-D, analysis revealed age related increased protein concentrations in the group between 60 and 90 years. All measured protein concentrations of the four SPs in samples of human articular cartilage are given in S2 Table.

### Distribution of all four SPs in cartilage tissue

**Fig 3:**
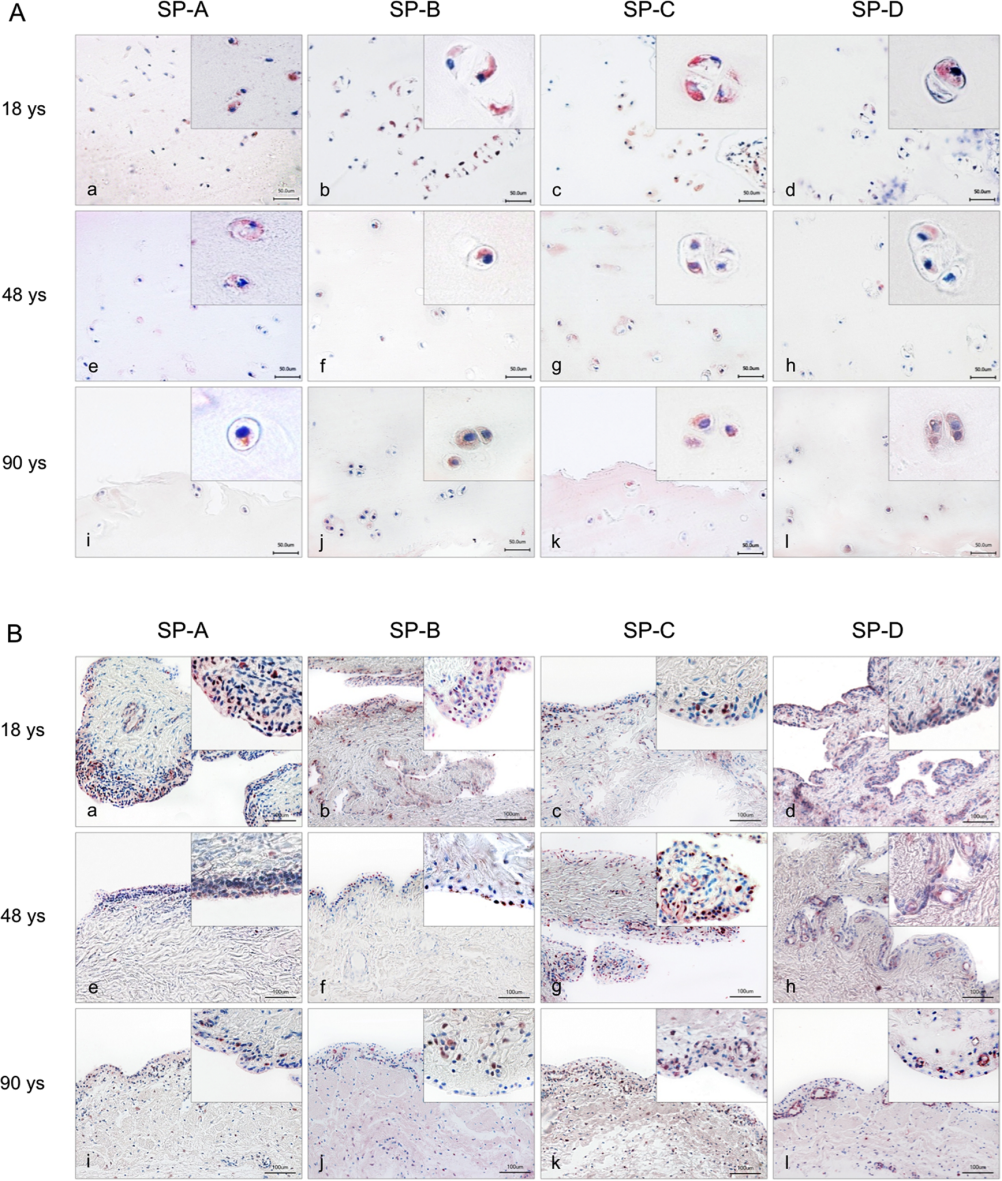
**Immunohistochemical detection (red staining) of surfactant proteins in chondrocytes of human articular joints (A) and synovial membrane (B). A:** Overview and detail screen of positive chondrocytes were represented in the tangential (superficial layer of cartilage) and transition zones (middle layer of cartilage) rather than the radial zone of articular cartilage. Samples from persons at age 18 years (a-d), 48 years (e-h) and 90 years (i-l) were used. **B:** Overview and detail screen of positive synoviocytes were represented in human synovial membrane. Samples from persons at the age of 18 years (a-d), 48 years (e-h) and 90 years (i-l) were used. Scale bars: 50 μm (A) and 100 μm (B).

**Fig 4:**
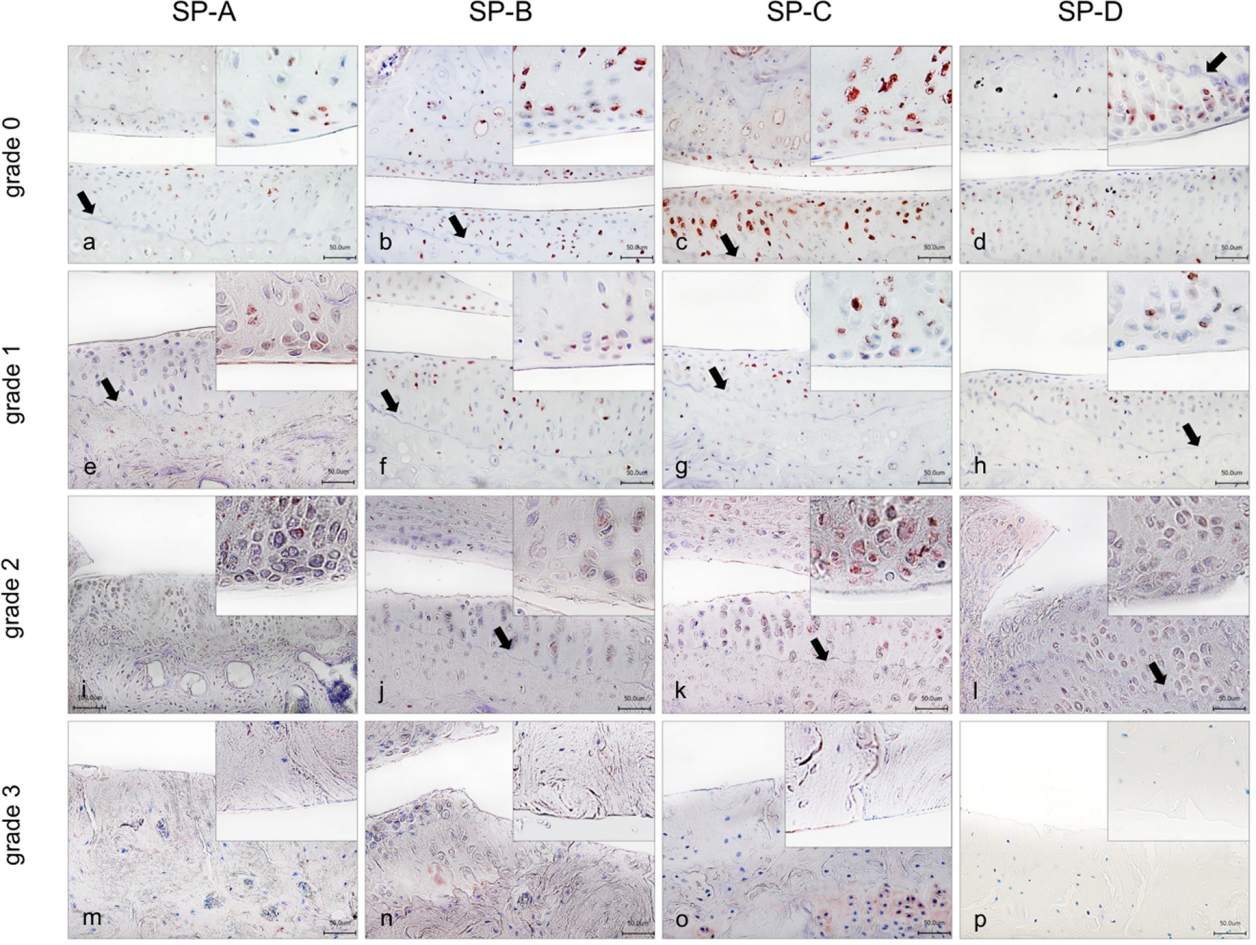
**Immunhistochemical detection of SP-A, -B, -C and -D in articular joint of** STR/ort **-mice.** Red staining indicates positive antibody reactions. Overview and detail screen of mice at 4 months with stage of OA 0°; 9 months with stage of OA I°, 10 months with stage of OA II° and 12 months with stage of OA III°. All surfactant proteins detected at the femur (F) and tibia plateau (T) with layer of cartilage, the joint line (J) and the tidemark (arrow). For SP-B, SP-C and SP-D stage of OA III° no cartilage. Scale bars: 50 μm.

SP localization was performed by immunohistochemical analysis of tissue sections from articular cartilage and synovial membrane obtained from patients aged 18, 48, and 90 years, as well as in tissue sections from knee joints of differently aged STR/ort mice (cf. Figs 3 and 4). Different patterns of positive antibody reactivity were visible in all samples. Immunohistochemical staining of human samples revealed immunostaining for all four SPs in chondrocytes of the tangential and transition zones rather than the radial zone of articular cartilage. No age-related difference in staining was apparent for any SP (Fig 3A).

Sections of synovial membrane revealed immune-reactivity for all four SPs in synoviocytes and weak staining in subsynovial tissue (cf. Fig 3B). Whether type A or type B synovial cells synthetize SPs was not analyzed in detail. Also, articular cartilage of STR/ort mice showed positive antibody reactivity for all four SPs (cf. Fig 4). STR/ort mice are characterized by an early onset of OA. Therefore, tissue slices at the age of 4, 9, 10 and 12 months that comply with OA degrees 0°, I°, II°, III° were used. SP-A was detected in chondrocytes of all analyzed ages in superficial layers of cartilage, as well as specifically below the tidemark (border between non-calcified and calcified cartilage) of the tibia. Immunohistochemical analysis of SP-B revealed immune-positivity mainly in chondrocytes of the layers above the tidemark. Only a few chondrocytes below the tidemark demonstrated positive immunostaining. SP-C-positive chondrocytes were located almost exclusively in the superficial cartilage layer of the tibia plateau. SP-D was found in chondrocytes of the tibia plateau, especially above the tidemark and only rarely below the tidemark.

### Influence of cytokines on SP expression by primary articular cartilage chondrocytes and chondrocytes of the immortalized cell line C28/I2

**Fig 5:**
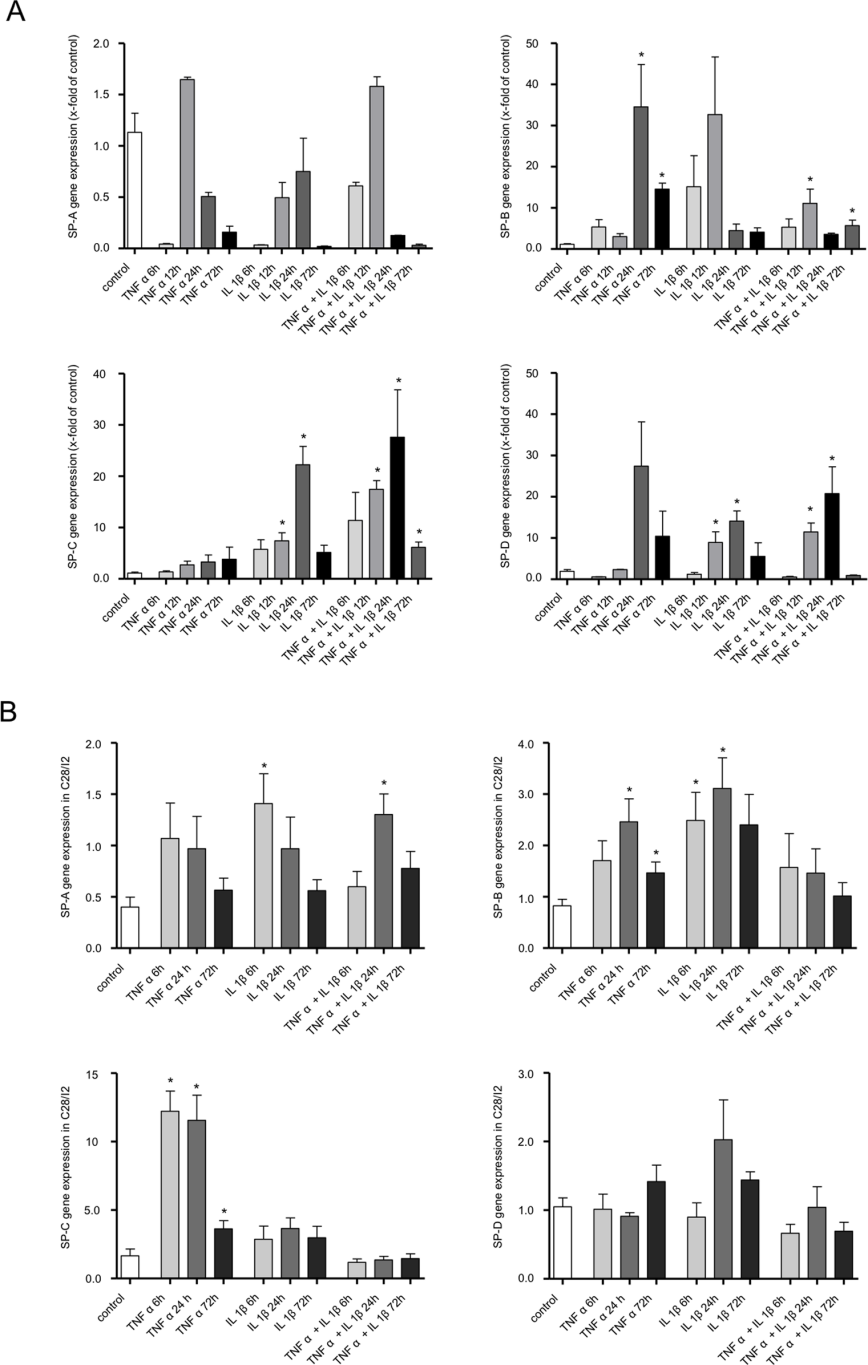
Relative SPs induction by inflammatory mediators/proinflammatory cytokines (10ng/ml tumor necrosis factor α [TNF α], 10ng/ml interleukin-1β [IL-1β]) quantified by real-time RT-PCR. **A:** Analysis of human primary chondrocytes. **SP-A** shows upregulation only at 12h and 24h TNF α, for IL-1β at 12h and 24h, in combination (TNF α + IL-1β) for 12h and 24h. **SP**-**B:** Significant upregulation is detected with TNF α for 24h and 72h and in combination with IL-1β for 12h and 72h. **SP-C:** Almost continuous stimulation time-dependent upregulation from 6h to 24h. Significant decrease is detected for 12 and 24h with IL-1β and in combination TNF α. **SP-D** Significant decrease is detected for 12 and 24h with IL-1β and in combination TNF α. Values are mean and SEM. Statistical significance: *P ≤ 0.05, **P ≤ 0.005, ***P ≤ 0.0005. **B:** Analysis of immortalized cells C28/I2 (n = 3). Expression of **SP-A, SP-B, SP-C** and **SP-D** at 6h, 24h and 72h compared to an untreated control. Values are mean and SEM. Statistical significance: *P ≤ 0.05, **P ≤ 0.005, ***P ≤ 0.0005.

To analyze the influence of cytokines on SP expression in human cartilage we performed experiments using primary chondrocytes and the human chondrocytic cell line (C28/I2) (cf. Fig 5A and 5B). Primary chondrocytes expressed low levels of mRNA for all four SPs without any treatment (cf. Fig 5A). Stimulation with TNFα or IL-1β alone, or a combination of both, did not significantly change SP-A mRNA. Stimulation with a combination of IL-1β and TNFα resulted in non-significant upregulation of SP-B mRNA and a significant upregulation of SP-C and SP-D mRNA for 12 and 24 hours (cf. Fig 5A).

In C28/I2 chondrocytes, cytokine treatments increased the levels of mRNA for all four SPs. However, the increases were significant for SP-A only after stimulation with IL-1β and in combination with TNFα. For SP-B a significant increase, only after TNFα or IL-1β stimulation alone. A increases were significant for SP-C only after stimulation with TNFα for 6 and 24 hours. Stimulation with IL-1β, TNFα, or the combination showed no significant upregulation for SP-D (cf. Fig 5B and S3 and S4 Tables).

## Discussion

It is well described that SPs are surface-active and have immunological functions in both innate and adaptive immune defenses. SP-A and SP-D regulate functions of a variety of immune cells, including dendritic cells, T cells, neutrophils and macrophages [21–23]. OA and RA share some features, including chronicity with progressive morphological changes of articular cartilage and inflammatory processes in the synovium and subchondral bone, although there are differences relating to the extent of tissue damage and the composition of immune cell types [24–26]

In the present study we comprehensively investigated the synthesis and regulation of SPs A-D in cells in healthy human articular knee cartilage and human synovial membrane, and in synovial fluid. Moreover, joints from STR/ort mouse model of spontaneous OA and synovial fluids from patients with OA and RA were also analyzed for the presence of all four SPs.

Our results revealed regular synthesis of all four SPs in all tissues under investigation and also in synovial fluid throughout all ages and in both sexes. This expression/synthesis pattern was confirmed at the mRNA and protein levels. Western blot analysis revealed SP-A at 60 kDa corresponding to a protein dimer. This synthesis pattern is comparable to SP-A in human nasal mucosa [16], tear film [27], testis [17] and is compatible with a premature dimer form of SP-A [15]. Occurrence of SP-A has already been described in synovial membrane [28], as well as in fibroblast-like stem cells of synovial membrane that were shown to synthesize SP-A [12]. The detected protein band of SP-B has a molecular weight of 40kDa consistent with a pro-form. Different pro-forms have been described in the past for SP-B. It is well known that SP-B is modified by post-translational processing into an active form (7kDa)[29]. Pro-forms at 25kDa were detected in club cells (formerly-known Clara cells) and at 35kDa in human tear fluid and tissues of the lacrimal system [29]. In the present study we detected a SP-B pro-form at 40kDa in human articular joint cartilage and synovial fluid. SP-C occurred in the respective tissues and synovial fluids at molecular weights of 16kDa and 26kDa in articular joint tissues and at 8kDa in synovial fluid. The active form of SP-C has a molecular weight of 4-6kDa, pro-forms were detected at 26kDa [30] and 21kDa [29] and intermediates at 16kDa and 7kDa [31]. SP-D was detected at a molecular size of 43kDa in all tissues investigated and in synovial fluid corresponding to the known monomer form of SP-D [15].

Immunohistochemical localization revealed cytoplasmic staining for all four SPs in chondrocytes of human articular cartilage of all investigated patients aged 18, 48, and 90 years. Of these, the lipophilic SPs (B and C) revealed immunoreactivity more in the tangential and transition zones than in the radial zone of articular cartilage. All four SPs were also detected in synovial membranes of all age groups. Our findings indicate that both type A (CD68+ macrophage-like) and type B (fibroblast-like synovial fluid-producing) synoviocytes are able to produce SPs. A clear separation, haven't been performed between the overlying synoviocyte layer and subintimal layer.

Functional aspects of SP-B and SP-C have been demonstrated in lung, auditory tube, and tear fluid [29, 32–34]. Both proteins are essential for the formation and stability of lipid layers and contribute to lowering surface tension.[35] In addition, SP-B and SP-C can regulate the permeability of phospholipid membranes [5]. Inherited defects of SP-B and SP-C lead to respiratory failure and interstitial lung disease in early childhood[36]. With regard to their ability to reduce surface tension, modulate permeability of biomembranes and change viscosity of phospholipid layers combination with plasmalogens and cholesterol [37], we speculate that SP-B and SP-C in diarthrodial joints are secreted by synoviocytes and articular chondrocytes into highly viscous synovial fluid, where they contribute to its rheological properties and facilitate and support convection into and out of articular cartilage.

Both SP-A and SP-D belong to the collectin family and show cytoplasmic distribution comparable to that of SP-B and SP-C in our present investigation. In lung, SP-A and SP-D show antimicrobial activity. Moreover, SP-A improves the SP-B mediated surface tension-reducing properties of surfactant lipids and maintains homeostasis between the extracellular and intracellular surfactant pools (for review see [38]). SP-D binds carbohydrates and lipids in a calcium-dependent manner and interacts with a number of microorganisms. Moreover, it binds to the core antigens in lipopolysaccharide in a manner distinct from SP-A binding (for review see [38]). Both SP-A and SP-D are present in the surfactant layer lining the alveolar surface, as well as within the apical portion of type II alveolar epithelial cells [39]. With regard to our present findings and the functions of SP-A and SP-D, we hypothesize that both proteins support the functions of the lipophilic SPs and are pathogen-binding proteins that support the immune defense system of articular joints against microbial invaders that can enter the joint mainly when circulating in the bloodstream. In this context, both type A and type B synoviocytes are able to produce a number of different antimicrobial peptides and proteins under healthy and inflamed conditions, contributing to innate and adaptive immune defenses in diarthrodial joints [40].

After evaluating the synthetic pattern of the four SPs in healthy joint structures throughout life, we assessed SPs in STR/ort a mouse model of OA. All four SPs were detected in chondrocytes of different stages of OA. This finding shows only that during OA at least no failure of SP production occurs and all 4 SPs are still produced. For several other proteins it has been shown that induction occurs in case of OA [8, 41]. However, during preparation of this manuscript [42] demonstrated in two rat models of OA that SP-D is produced by articular chondrocytes (confirming our result with regard to SP-D), with higher level in normal chondrocytes compared to in OA chondrocytes. Using recombinant SP-D the authors could demonstrate that SP-D ameliorates cartilage degeneration in a surgically-induced rat OA model by suppressing sodium nitroprusside-stimulated chondrocyte apoptosis and suppressing p38 MAPK activity [42].

Unfortunately, we had not enough material from all age groups and from STR/ort mice cartilage and synovial membrane to perform in addition quantification studies of all four SPs by ELISA to find out whether OA also is associated with up- or downregulation of SP production throughout live or during OA. This should be done in future studies and is a clear study limitation of the presented results. With regard to this it would also be very interesting to study the surfactant protein distribution qualitatively and quantitatively in the different cartilage zones.

To get further hints of a possible regulation of SPs in articular cartilage as this has been shown for antimicrobial peptides and other proteins in OA [8, 41] and as mentioned above for SP-D of rat artilcular chondrocytes [42], we analyzed this aspect further by means of cell culture experiments and quantitative methods.

To this end, we quantified all four surfactant proteins in synovial fluids harvested from patients with OA and RA and compared them to healthy human synovial fluids. Our ELISAs revealed significantly increased protein synthesis of all four SPs in human OA and RA synovial fluids.

Increased concentrations of SP-A and SP-D have also been detected in bronchoalveolar lavage of patients with inflammatory diseases like bronchial asthma [43] or allergic rhinitis [16], consistent with results of increased levels of SP-A and -D in RA synovial fluid [14]. Other studies of RA indicate that SP-D diffuses from the blood serum via the synovium depending on the degree of inflammation [13]. These findings are consistent with our results showing significant increases of lipophilic SPs SP-B and SP-C in OA and RA. Whereas SP-B showed anti-inflammatory activity in the lung [44], another study demonstrated significantly increased SP-B synthesis in chronic rhinosinusitis compared to healthy tissue [16]. However, impact on the disease process of the observed upregulated synthesis of all SPs in cases of OA and RA remains speculative and further investigations are required. Of special note is the finding that all four SPs are measurable in cadavers in significantly higher concentrations in “healthy” articular cartilage than in synovial fluid suggesting storage of SPs in articular cartilage or special functions of SPs inside articular cartilage. There is an increase of SP-A and SP-D with increasing age (group 60–90 years), which is at least significant for SP-D and reveals a clear tendency for SP-A. This is not obvious for the lipophilic SPs SP-B, and SP-C. Of interest, comparison of the three different age groups revealed a reduction of chondrocytes with increasing age although the total surfactant protein concentration in articular cartilage remains nearly constant for each SP under investigation in all three age groups (S2 Table). This finding suggests that either each chondrocyte in articular cartilage of aged people produces more surfactant protein (to reach the same total protein concentration than in the young age group) or a correlation exists between the surfactant protein concentration in articular cartilage and in synovial membrane. With other words if apotosis of chondrocytes occurs during aging the loss of surfactant protein production by chondrocytes is overtaken by synovial membrane. Of course a third possibility remains: chondrocytes in aged articular cartilage produce more SPs as do synoviocytes in synovial membrane in aged people. We speculate that the surfactant protein concentration is influenced by synovial membrane. However, this needs to be determined in future investigations.

The proinflammatory cytokines IL-1β and TNFα are key agitators of the catabolic events that can be observed in OA and in RA. Both cytokines are significantly upregulated in human OA [45], in STR/ort mice [46], and in human RA [47]. To analyze the effects of mediators of OA with regard to SP expression in articular cartilage, we cultured primary articular chondrocytes from OA patients, as well as C28/I2 chondrocytes (in order to have 2 different models and to study the usefulness of C28/I2 chondrocytes) and analyzed the expression patterns of SPs under basal conditions and after challenge with IL-1β and TNFα. Although some significant regulation was observed for cultured C28/I2 chondrocytes after stimulation with IL-1β or/and TNFα the x-fold upregulation on the x-axis was very low compared to primary chondrocytes (with the exception of SP-A) and in addition the effect was in most cases completely different to primary chondrocytes so that we conclude from these experiments that the C28/I2 cell line is not useful for the investigation of SP function in articular cartilage. One reason might be that the C28/I2 cell line is transfected with the large T antigen leading to changes in the response to external stimuli (cytokines). Similar differences between the responsiveness of cultivated primary chondrocytes and C28/I2 have also already been described for other proteins [8, 41]. After challenge with IL-1β, TNFα or in combination, primary articular chondrocytes from OA patients demonstrated partially a significant mRNA upregulation for SP-B, SP-C, and SP-D, but not SP-A. TNF-α and IL-1β seem to suppress the SP-A gene expression at 6 hours. SP-B was upregulated only by TNFα, whereas SP-C and SP-D were upregulated only by IL-1β. Combinations of IL-1β and TNFα increased only SP-C and SP-D. It was somewhat unexpected that SP-A mRNA in primary chondrocytes showed no regulation after cytokine treatment, since regulation of SP-A and SP-D, as already mentioned above, has been described in various tissues following bacterial colonization, inflammation, and autoimmune inflammatory processes [14, 48]. Whether SP-A and SP-D are also involved in the balance between matrix metalloproteases (MMPs) and tissue inhibitors of matrix metalloproteases (TIMPs) needs to be determined in further investigations. MMPs and TIMPs are essential for cartilage regeneration and undergo changes in the course of OA. This is of interest, since an imbalance between MMPs and TIMPs has also been described in human lung during inflammation for alveolar macrophages [49], for COPD patients during exacerbation [50] and in SP-D gene inactivated mice [51]. In addition, it is also possible that SP-D plays another role by taking part in remodeling processes of acute and chronic diseases in human fetal, newborn, and adult tissues [52]. And as already mentioned above data from rat cartilage suggest a protective role of SP-D by suppressing chondrocyte apoptosis and thus ameliorating cartilage degeneration. Further investigations with regard to all four SPs are needed to elucidate this issue.

In conclusion, our results show that surfactant proteins A, B, C, and D are physiological components of healthy articular cartilage, synovial membrane and synovial fluid, are upregulated in OA and RA and can be partly regulated on mRNA level by the proinflammatory cytokines IL-1β and TNFα. However, the latter statements are limited by the fact that we used “healthy” synovial fluid that was obtained from cadavers in most cases after a longer post mortem interval (10–14 h) than the synovial fluid from OA and RA patients with which it was compared (1–2 h) and that regulatory effects by cytokines were only studied on the mRNA and not yet on protein level. Thus our data are preliminary with regard to regulation of surfactant proteins, needing analysis in greater depth in further investigations.

## Supporting information

S1 Table ELISA: SPs synthesis in OA, RA and HS affected synovial fluid. Values are means.
(DOCX)

S2 Table ELISA protein concentration and cell count of young (0–30 years) -, middle-aged (30–60 years)—and elderly (60–90 years) persons. Values are means.
(DOCX)

S3 Table Real-time RT-PCR (primary chondrocytes): upregulations of protein concentration compared to control, which was normalized to 1. Values are means.
(DOCX)

S4 Table Real-time RT-PCR (immortalized cells): upregulations of protein concentration. Values are means.
(DOCX)
