# Supplementary material for: Evaluation of surfactant proteins A, B, C, and D in articular cartilage, synovial membrane and synovial fluid of healthy as well as patients with osteoarthritis and rheumatoid arthritis
Source: PLoS One. 2018 Sep 20;13(9):e0203502. doi: 10.1371/journal.pone.0203502 (PMC6147433; doi:10.1371/journal.pone.0203502)
Supplement: S1 Table — Values are means. (DOCX) [file pone.0203502.s001.docx]

**S1 Table:** ELISA: SPs synthesis in OA, RA and HS affected synovial fluid. Values are means.

| ELISA | ng/mg protein conc. in OA | ng/mg protein conc. in RA | ng/mg protein conc. in HS |
| --- | --- | --- | --- |
| SP-A | Ø 1.48 [0.7 – 2.7] | Ø 2.56 [0.9 – 6.8] | Ø 0.48 [0.09 – 0.97] |
| SP-B | Ø 2.82. [0.72 – 6.9] | Ø 9.38 [1.23 – 22.9] | Ø 1.96 [0.1 – 5.9] |
| SP-C | Ø 2.1 [0.9 – 3.2] | Ø 2.85 [1.1 – 4.0] | Ø 0.1 [0.01 – 0.4] |
| SP-D | Ø 0.61 [0.37 – 0.97] | Ø 0.77 [0.3 – 1.5] | Ø 0.23 [0.13 – 0.37] |
