# Supplementary material for: Evaluation of surfactant proteins A, B, C, and D in articular cartilage, synovial membrane and synovial fluid of healthy as well as patients with osteoarthritis and rheumatoid arthritis
Source: PLoS One. 2018 Sep 20;13(9):e0203502. doi: 10.1371/journal.pone.0203502 (PMC6147433; doi:10.1371/journal.pone.0203502)
Supplement: S2 Table — Values are means. (DOCX) [file pone.0203502.s002.docx]

**S2 Table**: ELISA protein concentration and cell count of young (0-30 years) -, middle-aged (30-60 years) - and elderly (60-90 years) persons. Values are means

| ELISA | 0-30 years | | 30-60 years | | 60-90 years | |
| --- | --- | --- | --- | --- | --- | --- |
|  | **conc. ng/mg** | **Cell count** | **conc. ng/mg** | **Cell count** | **conc. ng/mg** | **Cell count** |
| SP-A | 30.67 | 144 | 32.41 | 98 | 38.23 | 76 |
| SP-B | 22.47 | 144 | 20.03 | 98 | 20.29 | 76 |
| SP-C | 23.7 | 144 | 37.19 | 98 | 29.58 | 76 |
| SP-D | 23.62 | 144 | 20.82 | 98 | 35.59 | 76 |
