# Supplementary material for: Evaluation of surfactant proteins A, B, C, and D in articular cartilage, synovial membrane and synovial fluid of healthy as well as patients with osteoarthritis and rheumatoid arthritis
Source: PLoS One. 2018 Sep 20;13(9):e0203502. doi: 10.1371/journal.pone.0203502 (PMC6147433; doi:10.1371/journal.pone.0203502)
Supplement: S3 Table — Values are means. (DOCX) [file pone.0203502.s003.docx]

**S3 Table**: Real-time RT-PCR (primary chondrocytes): upregulations of protein concentration compared to control, which was normalized to 1. Values are means.

| Stimulations | 6h | 12h | 24h | 72h |
| --- | --- | --- | --- | --- |
| SP-A |  |  |  |  |
| TNF α | 0.02 | 3.76 | 27.1 | 0.16 |
| IL-1β | 0.02 | 0.50 | 0.75 | 0.01 |
| TNF α + IL-1β | 0.31 | 1.88 | 3.26 | 0.03 |
| SP-B |  |  |  |  |
| TNF α | 10.01 | 2.52 | 26.95 | 11.27 |
| IL-1β | 11.91 | 25.63 | 6.16 | 5.59 |
| TNF α + IL-1β | 5.30 | 11.11 | 5.03 | 4.82 |
| SP-C |  |  |  |  |
| TNF α | 1.35 | 2.13 | 3.27 | 2.87 |
| IL-1β | 5.74 | 7.42 | 16.59 | 15.35 |
| TNF α + IL-1β | 11.39 | 17.45 | 27.63 | 17.92 |
| SP-D |  |  |  |  |
| TNF α | 1.74 | 1.86 | 9.22 | 5.22 |
| IL-1β | 1.00 | 4.48 | 7.06 | 1.51 |
| TNF α + IL-1β | 2.03 | 3.62 | 13.92 | 14.50 |
