# Supplementary material for: Evaluation of surfactant proteins A, B, C, and D in articular cartilage, synovial membrane and synovial fluid of healthy as well as patients with osteoarthritis and rheumatoid arthritis
Source: PLoS One. 2018 Sep 20;13(9):e0203502. doi: 10.1371/journal.pone.0203502 (PMC6147433; doi:10.1371/journal.pone.0203502)
Supplement: S4 Table — Values are means. (DOCX) [file pone.0203502.s004.docx]

**S4 Table**: Real-time RT-PCR (immortalized cells): upregulations of protein concentration. Values are means.

| Stimulations | Control | 6h | 24h | 72h |
| --- | --- | --- | --- | --- |
| SP-A |  |  |  |  |
| TNF α | 0.72 | 0.99 | 1.00 | 1.26 |
| IL-1β | 0.72 | 1.37 | 1.62 | 0.69 |
| TNF α + IL-1β | 0.72 | 0.68 | 1.68 | 1.36 |
| SP-B |  |  |  |  |
| TNF α | 1.46 | 1.91 | 2.07 | 2.11 |
| IL-1β | 1.46 | 2.49 | 2.52 | 2.73 |
| TNF α + IL-1β | 1.46 | 1.73 | 1.89 | 1.44 |
| SP-C |  |  |  |  |
| TNF α | 1.37 | 2.1 | 1.1 | 2.7 |
| IL-1β | 1.37 | 1.6 | 1.6 | 2.7 |
| TNF α + IL-1β | 1.37 | 1.6 | 1.5 | 1.2 |
| SP-D |  |  |  |  |
| TNF α | 0.55 | 1.01 | 0.91 | 1.42 |
| IL-1β | 0.55 | 1.15 | 1.19 | 1.42 |
| TNF α + IL-1β | 0.55 | 0.66 | 0.61 | 0.69 |
